# Diagnostic Challenges and Insights in Optic Nerve Hemangioblastoma using Magnetic Resonance Imaging: A Case Report

**DOI:** 10.2174/0115734056359483250502063550

**Published:** 2025-05-15

**Authors:** Wenwen Wang, Fajin Lv, Tianyou Luo, Mengqi Liu

**Affiliations:** 1 Department of Radiology, First Affiliated Hospital of Chongqing Medical University, Chongqing 400016, China

**Keywords:** Optic nerve, Hemangioblastoma, Magnetic resonance imaging, Differential diagnosis, Case report, Von-Hippel Lindau, Heterogeneous hyperintensity

## Abstract

**Background:**

Optic nerve hemangioblastoma (ONH) is a rare benign tumor. It can be sporadic or associated with Von-Hippel Lindau (VHL) syndrome. Magnetic resonance imaging (MRI) is the most commonly used diagnostic technique for the tumor. However, an accurate diagnosis can be challenging due to the rarity of ONH and its similarity to glioma and meningioma.

**Case Report:**

A 49-year-old female experienced progressive vision loss for ten years in the right eye, accompanied by proptosis over two years. The ophthalmological examination found her visual acuity of the right eye to have no light perception. Optical coherence tomography showed decreased thickness of the right retinal ganglion cell layer. MRI revealed an oval solid mass within the right retrobulbar space, with isointensity on T_1_-weighted (T_1_WI) imaging and heterogeneous hyperintensity on T_2_-weighted imaging (T_2_WI). Heterogeneous enhancement was found on gadolinium-enhanced T_1_WI and dynamic contrast-enhanced MRI. At internal and marginal areas of the mass, multiple flow voids were observed on various sequences, especially on T_2_WI. Furthermore, the superior, inferior, medial, and lateral rectus muscles of the right eye distinctly atrophied, showing a lower signal intensity on T_2_WI and less apparent enhancement than the left normal ones. Postoperative pathological diagnosis was hemangioblastoma of the right optic nerve.

**Conclusion:**

Hemangioblastoma should be considered as a differential diagnosis for the space-occupying mass of the optic nerve if there is the presence of flow voids, vivid enhancement, and absence of a dural attachment, regardless of VHL syndrome. Of note, this is the first reported case to consider altered extraocular muscles as a potential point to prompt the diagnosis on MRI.

## 1 INTRODUCTION

Hemangioblastoma is a benign tumor of the central nervous system, accounting for 1-2% of all primary intracranial tumors, which predominantly occur in the cerebellum, medulla, and spinal cord [1, 2]. Supratentorial hemangioblastomas are exceedingly rare [3], and hemangioblastomas of the optic nerve, to our knowledge, have been reported in only 58 previous cases [1-27]. Common clinical symptoms in patients with optic nerve hemangioblastoma (ONH) include proptosis, headache, and vision loss to blindness, which can also be present in other optic nerve diseases. ONH can be sporadic (21%) or associated with Von-Hippel Lindau (VHL) syndrome (79%) [13, 22, 26], an autosomal dominant disease characterized by renal cell carcinoma, pancreatic cysts, and pheochromocytoma [28]. The illness has a serious impact on patients’ lives, especially blindness. It can be sharply demarcated from the adjacent nerve; therefore, it is potentially preventable when treated with a surgical approach aided by imaging guidance. Magnetic resonance imaging (MRI) is the most commonly used diagnostic technique [4]. Radiologists play a crucial role in the initial diagnosis and should be aware of this rare histological nature when called for a preoperative consultation of an optic nerve tumor. However, an accurate diagnosis can be challenging due to the rarity of ONH and its similarity to glioma and meningioma.

Herein, we report a case of ONH, in which the resultant vision loss was the only indication in a patient without VHL syndrome. Meanwhile, we review the multi-parameter MRI findings of the published cases of ONH, along with the associated clinical information, expecting to offer more imaging details to allow for accurate diagnosis and appropriate management.

## 2 CASE REPORT

A 49-year-old female experienced progressive vision loss for ten years in the right eye, accompanied by proptosis over two years. There was no complaint in the left eye. She had no other neurologic or general signs and symptoms. Her family history was unremarkable.

On ophthalmologic examination, the visual acuity of her right eye had no light perception and was 1.0 in the left eye. Exophthalmos was observed to be 15mm and 12mm, respectively, in the right and left eye. The orbital distance was 98 mm. The cornea was transparent in both eyes. The pupils were 2.5 mm and of the same size in both eyes. The crystalline lens was clear, and the vitreous body was nebulous. Optical coherence tomography (OCT) showed the thickness of the right retinal ganglion cell layer to be decreased. Additionally, the fundoscopic examination revealed no signs of optic disc edema or bleeding. Routine laboratory tests, including complete blood count and biochemical indices, were all within normal ranges.

**Fig. (1):**
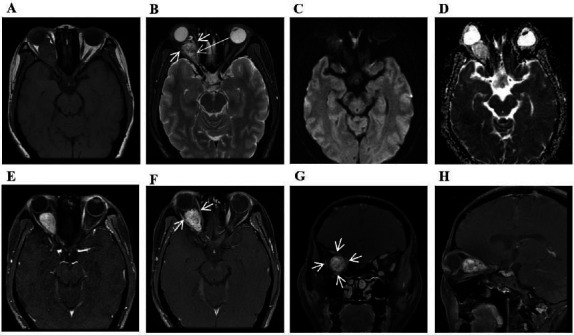
The mass showed isointensity on T_1_WI (**A**), heterogeneous hyperintensity with multiple flow voids (**long arrow**) on FS-T_2_WI (**B**), hypointensity on DWI (**C**), and hyperintensity on the ADC map (**D**). Heterogeneous enhancement was demonstrated on DCE-MRI (**E**) and Gd- FS-T_1_WI (**F-H**). A dilated vein was identified in the left anterior aspect of the mass (**B, E**). It should be noted that the superior, inferior, medial, and lateral rectus muscles of the right eye (**short arrows**) distinctly atrophied with a lower signal intensity on FS-T_2_WI (**B**) and less apparent enhancement than the left normal ones (**F, G**).

MRI revealed an oval solid mass occupying the right retrobulbar space, measuring 29mm×17mm×18mm, with isointensity on T_1_-weighted imaging (T_1_WI) and heterogeneous hyperintensity on fat-suppressed T_2_-weighted imaging (FS-T_2_WI), compared to brain parenchyma (Fig. **1A**, **B**). The mass showed hypointensity on diffusion-weighted imaging (DWI) (Fig. **1C**) and hyperintensity on apparent diffusion coefficient (ADC) maps (Fig. **1D**). Heterogeneous enhancement with a clear boundary was found on dynamic contrast-enhanced MRI (DCE-MRI) (Fig. **1E**) and gadolinium-enhanced fat-suppressed T_1_WI (Gd-FS-T_1_WI) (Fig. **1F**-**H**). At internal and marginal areas of the mass, multiple flow voids were observed on T_1_WI, FS-T_2_WI, DCE-MRI, and Gd-FS-T_1_WI, especially on FS-T_2_WI (Fig. **1B**). A dilated vein was also identified in the left anterior aspect of the mass (Fig. **1B**, **E**). The right intraorbital optic nerve could not be well distinguished. Furthermore, the superior, inferior, medial, and lateral rectus muscles of the right eye distinctly atrophied, showing a lower signal intensity on FS-T_2_WI (Fig. **1B**) and less apparent enhancement than the left normal ones (Fig. **1F**-**H**). Based on the MRI characteristics, the preoperative diagnosis was an optic nerve tumor, raising the suspicion of ONH.

**Fig. (2):**
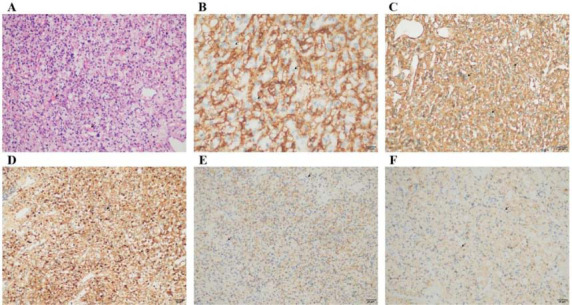
Histological features of the optic nerve hemangioblastoma (×200). Hematoxylin-eosin staining of the excised mass showed vacuolated stromal cells and numerous capillary vessels containing red cells. (**A**) Immunohistochemistry staining of the mass showed positivity for CD56 (**B**), vimentin (**C**), S-100 (**D**), NSE (**E**), and α-inhibin (**F**).

A resection of the right intraorbital tumor was performed while the patient was under general anesthesia. Intraope-ratively, the mass, approximately 2.8 cm in diameter and of a grey-white or grey-yellowish appearance, was noted to sur-round the optic nerve and was poorly demarcated. After surgery, the mass was removed successfully. Hematoxylin-eosin staining of the excised mass revealed vacuolated stromal cells along with numerous capillary vessels containing red blood cells, indicating active vascularization and potential neoplastic processes within the tissue. Immunohistochemistry staining showed positivity for CD56, vimentin, S-100, neuron-specific enolase (NSE), and α-inhibin, while negativity for epithelial membrane antigen (EMA, usually positive in renal cell carcinoma) and D2-40. Postoperative pathological diag-nosis was hemangioblastoma of the right optic nerve (Fig. **2**).

Five months after the operation, the patient’s clinical symptoms improved and there was no proptosis, but there was also no significant change in visual acuity.

## 3 LITERATURE REVIEW

While conducting the literature search for this study, we utilized several comprehensive databases to ensure a thorough review of the existing literature related to ONH. The primary databases searched included PubMed and Web of Science. The search was performed using a combination of the following keywords: 'optic nerve hemangioblastoma', 'magnetic resonance imaging', 'hemangioblastoma', and 'neuroimaging'. We limited our search to articles published in the English language from January 1992 to December 2023.

Hemangioblastomas of the central nervous system are uncommon vascular tumors characterized as benign, slow-growing, and non-metastasizing neoplasms, representing 1-2% of intracranial tumors [28, 29]. Supratentorial hemangio-blastomas are even rarer, with more than 90% occurring in the posterior fossa and distinctly unusual in the optic nerve [3]. Only 58 documented cases of the optic nerve have been found in the literature. Forty-one of them involved imaging studies, which significantly contributed to making a preoperative diagnosis, including angiography in nine patients [8, 9, 11, 15, 18], computed tomography (CT) in ten [4, 8, 18, 22, 23], and MRI in thirty-three [1-27]. The most sensitive imaging technique for detecting ONH has been found to be MRI, which was first performed on a patient with ONH in 1992 [4]. We performed a thorough review of the multi-parameter MRI findings on the published cases of ONHs along with the associated clinical information (Table **1**).

For all 33 cases of ONH, the age of the patients ranged from 12 to 64 years, including only three under the age of twenty. The male-to-female ratio was relatively equal. The most common symptom was vision loss in all but eight patients. Four were initially asymptomatic [9, 10, 16], and the other four had no reported clinical manifestation [5, 16]. Some patients with vision loss took the form of afferent pupillary defects [3, 7, 8, 14, 19, 22] and visual field defects [2, 3, 7, 8, 12, 13, 16, 17, 21]. Five other patients had headaches [2, 8, 20, 22, 27], while proptosis was reported in five patients [11, 13, 15, 20, 24]. Optic nerve pallor [4, 12, 15, 16, 21, 25, 27] and atrophy [2, 7, 17, 22] could be observed on ophthalmological examination. The course ranged from one week to twelve years, with only six patients experiencing symptoms for less than one year.

ONH occurs both sporadically and in association with VHL syndrome. At least twenty-five patients had VHL syndrome [3-10, 12, 14-16, 19-21, 23, 25-27], while the other eight exhibited no evidence of VHL syndrome [1, 2, 11, 13, 17, 18, 22, 24]. Other lesions of the central nervous system were observed in thirteen patients [3, 4, 6-10, 16, 19-21, 26, 27], involving tumors of the cerebellum, medulla oblongata, optic disc, and spinal cord. Retinal hemangioblastomas were presented in six patients [7, 10, 12, 16, 20]. In none of the patients was ONH considered to be an extension or metastasis from the original hemangioblastomas. Multiple hemangio-blastomas are thought to represent multifocal lesions [8, 30]. Eleven patients with VHL syndrome-associated lesions had multiple visceral cysts, pheochromocytoma, paraganglioma, and renal cell carcinoma [4, 6-9, 12, 16, 19, 21, 23, 27].

According to the MRI studies, most reported cases were unilateral, slightly more on the left side, and three had a bilateral presentation [4, 12, 19]. Seven tumors were limited to the orbit [3, 13, 16, 21, 23, 24], two were along the optic canal [16, 25], twelve were exclusively intracranial [4, 5, 7, 9, 10, 14, 16, 17, 27], and eleven involved the intraorbital and/or intracranial portions of the optic nerve, passing through the optic canal [1, 2, 6, 8, 11, 12, 15, 18, 20, 22, 26]. One case reported bilateral lesions involving the right intraorbital and the left intracranial optic nerve [19]. The size was usually not large, with a maximum diameter of 45mm recorded in the literature [15]. In general, they appeared to be morphologically regular and frequently fusiform or oval. Of 33 cases, twenty-four presented with solid tumors [4-9, 11-21, 23, 25-27], and only nine lesions had cystic components [1-3, 10, 16, 22, 24]. It was hard to distinguish between the tumors and the optic nerve on imaging, but the boundaries were well-demarcated from other surrounding structures. T_1_WI was available in ten cases, seven showed isointense masses [9, 11, 13, 17, 22, 24, 27] compared to the brain parenchyma, while three were hypointense [2, 16, 26]. Twelve cases that had T_2_WI showed hyperintense masses, homogenously or heterogeneously [2, 3, 9, 11-13, 16, 17, 22, 24, 26, 27]. Of them, the flow voids were observed at the internal and peripheral areas of the tumors in nine cases on T_2_WI [11, 13, 16-18, 22]. Advanced MRI techniques were also complementary in making a diagnosis. Arterial spin labeling (ASL) revealed increased perfusion in the tumor, which lighted up as a strawberry red spot [22]. The lesion showed evident hyperintensity on ADC maps but not on DWI, compared to brain parenchyma [26]. On contrast-enhanced images, significant enhancement was observed, homogenously in seventeen [4, 6-10, 12-16, 19, 21, 22, 25-27] and heterogeneously in ten cases [1-3, 11, 16-18, 20, 23, 24]. Eleven cases displayed the surrounding edema, which coursed along the interconnected white matter tracts of the optic system [2-4, 14, 16, 18, 20, 24, 26]. Bilateral frontal lobe edema was also present. However, edema may subside after surgery, potentially resulting in an improvement in vision [2-4, 14, 18, 20, 24, 26].

Macroscopically or histologically, the tumor-nerve interfaces were well-demarcated [2, 4, 8-10, 14, 16, 17, 25, 26]. Among the seventeen cases with surgical data, eleven involved the periphery of the optic nerve [1, 3, 9-11, 16-19, 26, 27], four encased the optic nerve [2, 7, 8, 25], and two were described as ‘splitting’ the optic nerve [4, 14]. Resected lesions had no involvement of the leptomeninges and no discernible tumor capsule [16, 22, 31, 32].

## 4 DISCUSSION

ONHs are extremely rare. Only 58 cases have been reported since the 1940s. They can occur in patients of any age group, but they less commonly present in those under twenty years of age. There are no significant gender differences. Common clinical manifestations include vision loss, headache, and proptosis, which are slowly progressive from onset. As we have reviewed, about 79% of patients with ONH can be associated with VHL syndrome. However, these symptoms can also be present in other kinds of optic nerve tumors, such as glioma and meningioma [21, 24]. Therefore, imaging studies, especially MRI, can play a pivotal role in making a preoperative diagnosis and guiding the subsequent treatment, although a definitive diagnosis of ONH can only be made by a pathological examination.

On imaging, most cases of ONH are unilateral and located in the pre-chiasmal optic nerve, including the intraorbital, intracanalicular, and intracranial segments [25]. The size is usually not large due to the limited orbital location, with a maximum diameter of 45mm recorded in the literature [15]. Generally, they appear to be morphologically regular and frequently fusiform or oval. The classic hemangioblastoma is a large cyst with intensely enhancing nodules [2]. However, hemangioblastomas of the optic nerve are mainly solid with relatively rare cystic components. The solid appearance might be linked to the unique location of the optic nerve, which could influence tumor morphology. While classic hemangioblastomas often exhibit cystic formations due to VHL gene mutations leading to abnormal vascular proliferation and subsequent fluid accumulation, the optic nerve’s confined anatomical space may restrict cystic expansion [33]. It is hard to distinguish the tumors and the optic nerve on imaging, but the boundaries are well-demarcated from other surrounding structures. MRI shows isointensity or hypointensity on T_1_WI while homogenous or heterogeneous hyperintensity on T_2_WI. On contrast-enhanced scans, prominent enhancement is demonstrated homogenously or heterogeneously with an absence of dural attachment [2]. At internal or peripheral areas of the tumor, irregular-shaped flow voids may be observed, especially on T_2_WI. Moreover, marked surrounding edema is also suggestive of ONH, which can propagate from the optic nerve to the optic chiasma, optic radiation, and even the contralateral optic nerve [2, 14, 16]. Bilateral frontal lobe edema can also be involved [24]. Previous studies have shown the surrounding edema of the central nervous system to be formed by the extravasation of plasma ultrafiltrate through leaky tumor vessels [14, 34], and the propagation of edema occurs preferentially along the low-resistance pathways, such as parallel fibers of white matter tracts [35-37]. These factors may explain why some unilateral lesions can lead to the impairment of bilateral vision [14, 26]. Even though hypervascularity can result in severe edema, intracranial or intratumoral hemorrhage has not been observed in supratentorial hemangioblastomas [16].

Flow voids and heterogeneous enhancement are of vital importance in diagnosing ONH, indicating abundant blood supply in the tumor, which is crucial for differentiating it from similar tumors, like gliomas and meningiomas in the optic nerve region. However, these features are not consistently reported across all studies, which may be related to the stage of tumor development or differences in imaging techniques. The absence of typical imaging features can make the diagnosis complicated. In such cases, more advanced imaging sequences and techniques can be beneficial, like DCE-MRI to capture subtle blood flow supply. Additionally, combining clinical presentation with other imaging features, such as marked surrounding edema, may serve as supplementary diagnostic indicators. Given the limitations of relying solely on MRI, it is suggested to integrate other imaging technologies, like CT angiography and optic nerve ultrasound, in complex cases to comprehensively assess eye structures and tumor blood supply.

In our case, the patient was a 49-year-old female who experienced progressive vision loss in her right eye over ten years. The MRI findings were consistent with general studies. Notably, multiple flow voids were apparent on FS-T_2_WI at internal and marginal areas of the mass. We observed significant atrophy in the superior, inferior, medial, and lateral rectus muscles of the right eye, with lower signal intensity on FS-T_2_WI and less apparent enhancement compared to the normal left ones. To our knowledge, these imaging features in patients with ONH have not been reported previously. This MRI presentation might be associated with the ‘steal syndrome’ phenomenon, where the tumor, due to its high vascularity, diverts blood supply from the surrounding normal tissues, potentially leading to reduced blood flow and subsequent muscle atrophy [1, 38, 39]. Additionally, other factors that could cause extraocular muscle atrophy should be considered, such as disuse atrophy, compression atrophy, and denervation atrophy [40]. After reviewing previously reported cases, we found one case with similar features that had not been described by the authors [15]. These changes could serve as indirect signs for diagnosing ONH, which might aid in the earlier detection of such lesions in clinical practice.

**Fig. (3):**
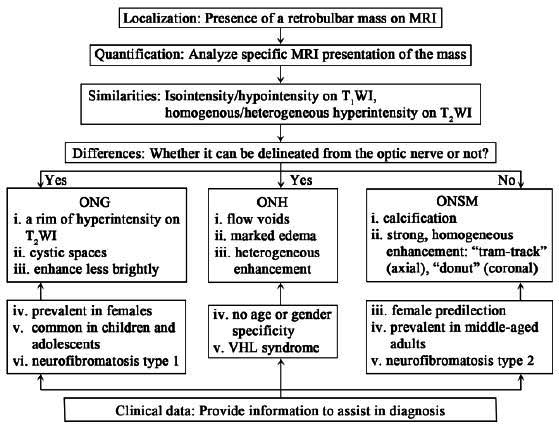
A flowchart summarizing the differential diagnosis for optic nerve tumors.

Considering the location in the optic nerve and MRI findings, optic nerve glioma (ONG) and optic nerve sheath meningioma (ONSM) are commonly considered differential diagnoses of ONH. In this work, we have drawn a flowchart to visualize the analytical process of differential diagnosis, based on MRI features and clinical information (Fig. **3**).

ONH is common in children under eight years old and occurs more frequently in females [41-43]. Of these, 45%-70% can involve chiasmal and post-chiasmal optic nerve [44, 45]. Gliomas arising from the optic nerve are difficult to delineate from the nerve itself on imaging, like hemangioblastomas [45]. However, gliomas show some specific imaging features. First, a rim of hyperintensity can be observed on T_2_WI, a finding that may mimic an expanded subarachnoid space [46]. Second, cystic spaces may be seen rather than flow voids [44, 46, 47]. Third, in contrast-enhanced studies, gliomas usually enhance less brightly than hemangioblastomas. Additionally, they can present with non-enhancing solid/cystic components [26]. Of note, optic tract edema is uncommon in gliomas [2, 48].

Moreover, meningioma is also considered a differential of ONH. Optic nerve meningiomas show female predilection (61–84%) and primarily affect middle-aged adults with a peak incidence between 45 and 55 years [49-51]. They locate slightly more in the right optic nerve (55–71%), while bilateral involvement (5%) can result from tumor extension along the optic canal and into the optic chiasm to the contralateral side [50-52]. Meningiomas arise from the surrounding optic nerve sheath and can be distinguished from the nerve at the coronal position on MRI [13]. Meanwhile, meningiomas show some other specific imaging features. First, calcification can be observed in 20%-50% of cases [46]. Second, flow voids are only seen in relatively large meningiomas [16]. Third, optic nerve meningiomas are well-enhanced. Because the nerve itself is spared, a ‘tram-track’ appearance is often observed at axial contrast-enhanced MRI, which is characterized by enhancement of meninges lying on either side of the hypointense nerve [51]. In coronal images, it appears as a donut [53].

The flowchart visualizes the analytical process of differential diagnosis, expecting to provide a clear approach to the diagnosis of optic nerve tumors, based on MRI features and clinical information.

While conventional MRI remains the cornerstone in the evaluation of optic nerve tumors, it is often insufficient for making a definitive differential diagnosis [26]. Although a few case reports have described the application of advanced imaging techniques, such as ASL, in detecting hypervascular characteristics of ONH [22, 26], their diagnostic specificity in distinguishing ONH from other optic nerve tumors remains unclear. Therefore, future research should consider incorporating more advanced and quantitative MRI techniques, including perfusion-weighted imaging, diffusion tensor imaging, and functional MRI parameters, to improve the radiological distinction. These may offer novel biomarkers that can assist radiologists in making more accurate preoperative diagnoses.

In the present case, surgical resection was performed, which remains the standard and most definitive treatment for ONH when anatomically and clinically feasible [22, 25, 26]. However, potential alternative treatment strategies, such as stereotactic radiotherapy and anti-angiogenic therapy, may be considered in reported cases [21, 24], particularly those associated with VHL syndrome or when surgical resection poses significant risks.

In addition, long-term follow-up is crucial to understand the prognosis of patients with ONH. Regardless of the presence or absence of VHL syndrome, periodic imaging, ophthalmologic examinations, and genetic counseling could still be pivotal. ONHs are usually in proximity to vital structures in the eye and brain, so complete removal of the tumor may be complicated. It is important to highlight that ONH, though rare, can recur.

## CONCLUSION

Hemangioblastoma should be considered as a differential for the space-occupying mass of the optic nerve if there is the presence of flow voids, vivid enhancement, and absence of a dural attachment, regardless of VHL syndrome. Of note, this is the first reported case to consider altered extraocular muscles as a potential point to prompt the diagnosis on imaging. ONH should be mainly differentiated from glioma and meningioma.

## AUTHORS’ CONTRIBUTIONS

W.W.: Writing the paper; F.L., T.L., M.L.: Writing, Reviewing and editing the paper.

## Figures and Tables

**Table 1:** Multi-parameter MRI findings of 33 published cases of optic nerve hemangioblastoma along with the associated clinical information.

**Author** **(Year)**	**Age (yr)/Sex**	**Symptoms and Signs**		**MRI**								**Von Hippel-Lindau Syndrome**
				**Location**	**Size** **(mm)**	**Cystic/** **Solid**	**T_1_**	**T_2_**	**Flow Voids**	**Enhanced**	**Other Findings**	
Ginzburg (1992) [4]	44/M	Progressive vision loss in the both eyes for 8 months; bilateral optic disc pallor		R/L; IC	NA	Solid	NA	NA	NA	Homo	Edema of bilateral white matter	Yes, pancreatic, hepatic, and renal cysts, RCC, tumors of cerebellum and medulla oblongata, negative family history
Miyagami (1994) [5]	26/F	NA		R; IC	10	Solid	NA	NA	NA	NA	NA	Yes
Rubio (1994) [6]	43/F	Progressive vision loss for 4 years; absence of the right pupillary light reflex and visual evoked response		R; IO/ICa	20×8	Solid	NA	NA	NA	Homo	NA	Yes, pancreatic, renal, and adnexal cysts, tumors of cerebellum, negative family history
Balcer (1995) [7]	21/F	Progressive vision loss for 2 years; visual field defect; afferent pupillary defect; atrophy of the left optic nerve		L; IC	18×13×22	Solid	NA	NA	NA	Homo	NA	Yes, hepatic and renal cysts, tumors of cervicomedullary junction and retina, negative family history
Kerr (1995) [8]	40/F	Progressive vision loss for 1 year; headache; visual field defect; afferent pupillary defect		R; ICa/IC	NA	Solid	NA	NA	NA	Homo	NA	Yes, tumors of cerebellum and right optic disc, pheochromocytoma, paraganglioma, RCC, positive family history
Raila (1997) [9]	30/F	Asymptomatic		L; IC	10×8×8	Solid	Iso	Hyper	NA	Homo	NA	Yes, tumors of right optic disc, pancreatic cysts, positive family history
Kouri (2000) [10]	15/F	Asymptomatic		L; IC	14×7	Cystic/ Solid	NA	NA	NA	Homo	NA	Yes, tumors of cerebellum and retina
Kato (2004) [11]	29/M	Progressive vision loss to blindness for 7 years; proptosis with dull pain; absence of the right pupillary light reflex		R; ICa/IC	NA	Solid	Iso	Hyper	Yes	Heter	NA	No, negative family history
Fons Martinez (2006) [12]	35/M	Progressive vision loss in the both eyes for 5 years; visual field defect in the left eye; bilateral optic disc pallor		R/L; ICa/IC	NA	Solid	NA	Hyper	NA	Homo	NA	Yes, retinal hemangioblastoma, renal cysts, pheochromocytoma, positive family history
Higashida (2007) [13]	64/M	Progressive vision loss for 5 years; proptosis; visual field defect; absence of the left pupillary direct light reflex		L; IO	NA	Solid	Iso	Hyper	Yes	Homo	NA	No, a renal cyst, negative family history
Baggenstos (2008) [14]	62/M	Near complete vision loss in the left eye for 1 week; vision loss and afferent pupillary defect in the right eye		L; IC	NA	Solid	NA	NA	NA	Homo	Edema of optic chiasm, bilateral optic nerves, bilateral geniculate bodies, and optic radiations	Yes
Barrett (2008) [15]	47/M	Vision loss for 6 years; proptosis; optic nerve pallor		R; IO/ICa	45	Solid	NA	NA	NA	Homo	NA	Yes
Meyerle (2008) [16]	60/F	Vision loss for 4 years; visual field defect; optic nerve pallor		R; IO	18×20×21	Internal cyst	Hypo	Hyper	Yes	Hetero	NA	Yes, pheochromocytoma, tumors of right orbit, cerebellum, spine cord, and pancreas
	15/F	Asymptomatic		L; IO	9×14	Solid	NA	NA	Yes	Homo	NA	Yes, retinal hemangioblastoma
	54/M	Vision loss		L; ICa	7×10	Cystic/solid	NA	NA	NA	Enhanced	Edema of optic chiasm and bilateral optic tracts	Yes
	29/F	Asymptomatic		L; IC	10×8×8	Solid	NA	NA	NA	Enhanced	NA	Yes, retinal hemangioblastoma, positive family history
	NA/M	NA		L; IC	16×8	Solid	NA	NA	NA	NA	NA	Yes
	NA/M	NA		R; IC	23×25	Internal cyst	NA	NA	Yes	NA	Edema of bilateral optic tracts	Yes
	NA/M	NA		R; IC	16×15	Internal cyst	NA	NA	Yes	NA	Edema of bilateral optic tracts	Yes
Prabhu (2009) [17]	32/M	Progressive vision loss for 8 months; visual field defect; primary optic atrophy		R; IC	35	Solid	Iso	Hyper	Yes	Hetero	NA	No, negative family history
Shima (2011) [18]	33/M	Blindness for 12 years		L; ICa/IC	26×18×22	Solid	NA	NA	Yes	Hetero	Edema of left optic tract	No, negative family history
Zywicke (2012) [1]	50/F	Progressive vision loss for 2 years; fatigue; dry skin; brittle nails; thinning hair		L; ICa/IC	10×10	Cystic/solid	NA	NA	NA	Hetero	NA	No
Fard (2014) [19]	39/M	Progressive vision loss in both eyes; afferent pupillary defect		R/L; IO/IC	NA	Solid	NA	NA	NA	Homo	NA	Yes, pheochromocytoma, tumors of optic disc and cerebellopontine angle area, negative family history
Staub (2014) [20]	34/F	Blindness; retro-orbital pain; proptosis		R; ICa/IC	12	Solid	NA	NA	NA	Hetero	Edema of bilateral optic tracts	Yes, tumors of cerebellum, craniocervical junction, cauda equina and retina, positive family history
Turel (2017) [2]	67/M	Progressive vision loss for 1 year; seeing halos around objects; headache; visual field defect; Primary optic atrophy		L; ICa/IC	18	Cystic/ Solid	Hypo	Hyper	NA	Hetero	Edema of bilateral optic tracts, more on the left side	No, negative family history
McGrath (2018) [3]	25/F	Progressive vision loss for 4 years; visual field defect; afferent pupillary defect		R; IO	3×8	Cystic/ Solid	NA	Hyper	NA	Hetero	Edema of right pre-chiasmal optic nerve, optic chiasm, and bilateral optic tracts	Yes, tumors adjacent to the fourth ventricle and in the spinal cord, positive family history
Kanno (2018) [21]	36/F	Progressive vision loss; visual field defect; optic disc pallor		L; IO	NA	Solid	NA	NA	NA	Homo	NA	Yes, renal and pancreatic cysts, tumors of cerebellum and spinal cord, positive family history
Darbari (2019) [22]	33/F	Progressive vision loss for 3 months; headache; optic atrophy; afferent pupillary defect		R; IO/ICa/IC	NA	Cystic/ Solid	Iso	Hyper	Yes	Homo	NA	No, negative family history
Boratto (2020) [23]	49/F	Vision loss for 7 years; rotational vertigo		L; IO	8×5×4	Solid	NA	NA	NA	Hetero	NA	Yes, renal and pancreatic cysts, RCC, positive family history
Xu (2020) [24]	51/F	Progressive vision loss for 1 month; proptosis		L; IO	NA	Cystic/ Solid	Iso	Hyper	NA	Hetero	Edema of optic chiasm, left optic tract, and bilateral frontal lobes	No
Alvarez (2021) [25]	55/M	Progressive vision loss; optic nerve pallor		L; ICa	5.3×4	Solid	NA	NA	NA	Homo	NA	Yes
Duan (2021) [26]	41/M	Progressive vision loss to blindness in the left eye for 6 years; reduced vision acuity in the right eye for 3 months		L; IO/ICa/IC	15	Solid	Hypo	Hyper	NA	Homo	Edema of left intraorbital optic nerve	Yes, tumors of cerebellum, negative family history
Yang (2021) [27]	12/M	Vision loss for 1 month; headache; dizziness; optic nerve pallor		L; IC	20×19×24	Solid	Iso	Hyper	NA	Homo	NA	Yes, tumors of cerebellum, pancreatic cysts, positive family history

**Abbreviations:** NA = Not available; M = Male; F = Female; R = Right; L = Left; IC = Intracranial; ICa = Intracanalicular; IO = Intraorbital; Iso = Isointensity; Hypo = Hypointensity; Hyper = Hyperintensity; Homo = Homogenous; Heter = Heterogeneous; RCC = Renal cell carcinoma

## Data Availability

All data generated or analyzed during this study are included in this published article.

## LIST OF ABBREVIATIONS

ONH: Optic nerve hemangioblastoma
VHL: Von-Hippel Lindau
MRI: Magnetic resonance imaging
OCT: Optical coherence tomography
T_1_WI: T_1_-weighted imaging
FS-T_2_WI: Fat-suppressed T_2_-weighted imaging
DWI: Diffusion-weighted imaging
ADC: Apparent diffusion coefficient
DCE-MRI: Dynamic contrast-enhanced MRI
CT: Computed tomography
ONG: Optic nerve glioma
ONSM: Optic nerve sheath meningioma

## STANDARDS OF REPORTING

CARE guidelines were followed.
